# Improving children’s ability to remember intentions: a literature review on strategies to improve prospective memory during childhood

**DOI:** 10.1007/s00426-023-01834-8

**Published:** 2023-05-25

**Authors:** Milvia Cottini

**Affiliations:** Cognitive and Educational Sciences (CES) Lab, Faculty of Education, Free University of Bolzano-Bozen, Regensburger Allee 16, 39042 Bressanone-Brixen, Italy

## Abstract

Children often fail to remember executing intentions because prospective memory (PM) does not completely develop until late adolescence or young adulthood. PM failures are often observed in children and can have negative consequences on their everyday lives. Thus, in the last 50 years, various strategies to support children’s PM have been designed and evaluated, such as prompting children to use different encoding modalities, such as verbal, visual, and enacted modalities, or encoding strategies, such as implementation intentions, episodic future thinking (EFT), and performance predictions, as well as providing children with verbal and visual reminders. However, not all these interventions have shown to efficiently enhance PM performance during childhood. The present literature review is aimed at summarizing these interventions and critically examining their effectiveness from a developmental perspective and by considering underlying mechanisms. The type of PM task (event-, time-, and activity-based), cognitive resource demands, and processing overlaps are also considered. Finally, directions for future research and possible applications in everyday life will be discussed.

## Introduction

Usually, adults can accomplish a previously formed plan, either by remembering on their own or by adopting strategies that help them remember; by contrast, children frequently struggle with these tasks (Mahy et al., 2014). The ability to remember and execute intended actions, defined as prospective memory (PM; Einstein & McDaniel, 1990), does not fully develop until late adolescence or young adulthood and children often rely on adults’ support (Kretschmer-Trendowicz & Altgassen, 2016; Zimmermann & Meier, 2006). Notwithstanding, at the start of primary school, children are gradually expected to remember intentions independently (Hajdas et al., 2021) and are often judged negatively by adults when they fail to do so (Moeller et al., 2021). School-aged children who frequently fail to remember are likely to be disadvantaged in various contexts of their life: in school, when they forget to do their homework or to complete assignments; in their relationships, when they forget to return a toy to a friend or to convey messages to their parents, and regarding their health and well-being, when they forget to wear a helmet when bike riding or to take the lunchbox to school (Mahy et al., 2014). Investigators have recognized the necessity of identifying ways to support prospective remembering during this developmental period (Kretschmer-Trendowicz et al., 2016). During the past 50 years, various strategies have been evaluated, such as presenting children with different encoding modalities (Li & Wang, 2015; Passolunghi et al., 1995), encouraging children to use an encoding strategy, such as implementation intentions, episodic future thinking (EFT), or performance predictions (Cottini et al., 2018; Kretschmer-Trendowicz et al., 2016, 2021), as well as providing children with visual or verbal reminders (e.g., Kliegel & Jäger, 2007; Mahy et al., 2018). Nevertheless, not all these strategies have shown to be effective during childhood. Moreover, these interventions have been evaluated primarily using laboratory-based experiments, whereas empirical studies on their usefulness in natural settings are lacking.

This review aims at summarizing the literature on PM interventions and critically examining their effectiveness in improving PM performance across childhood. First, PM task types, PM development, and underlying processes will be briefly described. Second, strategies to improve children’s PM will be presented, and studies on their effectiveness will be reviewed. Their impact will be examined from a developmental perspective and by considering PM task characteristics such as resource demands and processing overlaps as well as examining underlying mechanisms. Finally, the usefulness and application of these interventions in natural settings and suggestions for future research in this field will be discussed.

## Measuring prospective memory in children

PM has been primarily tested in laboratory-based experiments in which children are engaged in a task such as sorting cards (i.e., ongoing task) and are concurrently asked to remember to execute an embedded PM task (Kvavilashvili et al., 2001). Importantly, in these laboratory-based PM tasks, PM targets are rarely presented during the ongoing task (Brandimonte et al., 2001), thereby mimicking everyday-life PM tasks in which occasions to perform an intention are rare. Moreover, particularly in investigating PM in children, control questions are included at the end of the task to ensure children’s understanding and remembering of the PM task instructions and content (Kvavilashvili et al., 2008). The PM task can consist in, for example, carrying out a different action whenever a specific event occurs, such as separating off the cards that depict an animal (i.e., event-based), carrying out a specific action after a definite amount of time has passed (i.e., time-based), or performing a predefined action when the ongoing task is finished (i.e., activity-based; Einstein et al., 2008). In the real world, these tasks might correspond to, for example, buying butter when passing by the grocery store, taking cupcakes out of the oven after 10 min, or making a phone call after a lesson, respectively. Event-based PM tasks can differ in the degree of overlap between the PM and the ongoing tasks (Einstein & McDaniel, 2005; McDaniel et al., 2015; Meier & Graf, 2000; Meiser & Schult, 2008). For instance, an event-based PM task can be highly overlapping or focal with the ongoing task when both tasks require the same processing (e.g., semantic elaboration for both the ongoing task and the identification of PM targets). By contrast, an event-based PM task can be low overlapping or non-focal when the two tasks require different processing (e.g., semantic elaboration for the ongoing task and perceptual elaboration for the identification of PM targets; Maylor et al., 2002; Meier & Graf, 2000). A further distinction has been drawn concerning the specificity of the PM cue that can be well-specified, such as responding to the word *cat*, or categorical, such as responding to a word belonging to the animal category (Hicks et al., 2005; Meier & Cottini, 2023).

## Theoretical frameworks of prospective memory and its development

The two main theories which have guided PM research over the past few years attempt to explain the nature of PM retrieval (recently reviewed in Rummel & Kvavilashvili, 2022). For instance, according to the *multiprocess framework*, successful PM retrieval can occur via two pathways: top-down or bottom-up. Consequently, the environment can be strategically monitored to detect the PM targets (i.e., top-down), or the PM targets can spontaneously trigger intention retrieval (i.e., bottom-up). For instance, when the PM targets are easy to identify, such as with focal or salient PM cues, or when a strong cue–intention association is present, retrieval occurs automatically without effortful control. In contrast, when the PM targets are non-focal or non-salient, or when the PM task is time-based or important, retrieval relies on top-down attentional resources (McDaniel et al., 2015). Effortful control can also be engaged in response to the context in which PM targets are anticipated. In addition to the non-focal/focal distinction, the *process overlap framework* (Meier & Graf, 2000) suggests that performing high overlapping or focal PM tasks can require some cognitive resources when they include a parallel overlap (i.e., categorical PM cue) compared to a parallel and sequential overlap (i.e., specific PM cue; Meier & Cottini, 2023). Finally, a more recent extension of the multiprocess framework has proposed that PM retrieval may be a dynamic process, with a dynamic interaction between strategic and automatic retrieval processes throughout the PM task (Shelton & Scullin, 2017).

In contrast, the *preparatory attention and memory* (PAM) theory (Smith, 2003; Smith & Bayen, 2004) holds that attentional resources are always needed to perform a PM task successfully. Correspondingly, preparatory attention processes are required to allocate attention away from the ongoing task toward the PM task, and to constantly keep the intention in working memory (WM). Because WM resources are limited and shared between the ongoing task and the PM task, successfully performing the PM task interferes with the ongoing task by producing a cost. Attentional monitoring costs are characterized by slower ongoing task RTs or lower ongoing task accuracy rates; these aspects are assumed to be consistent with the idea that the ongoing task and the PM task rely on the equivalent limited WM capacity (Smith & Bayen, 2005). In response to criticism regarding the constant reliance on WM resources, the PAM theory has recently been extended through inclusion of a context-dependency hypothesis, according to which preparatory attention processes are activated only in response to the context in which the PM targets are anticipated to appear (Smith, 2017).

On the basis of these two main models of adult PM, Mahy et al. (2014) have described a specific model to explain age-related improvements in PM during childhood: the *executive framework* of PM development. The authors theorized that, in addition to retrospective memory, executive processes such as WM, inhibition, switching, planning, and attentional monitoring would be the major driving forces of PM development during childhood. While in early childhood, retrospective memory is mainly responsible for children’s PM difficulties (i.e., children frequently forget what they have to do), starting from the age of 4 years, children’s PM failures appear to depend more on executive errors (i.e., children frequently forget to perform the PM task even though they remember what they had to do). Along these lines, the age-related variations in PM performance would be more evident for non-focal and time-based versus focal PM tasks. The different executive processes have been suggested to be more or less relevant at different ages. Accordingly, WM would be more relevant during early childhood, whereas inhibition, attentional monitoring, and switching would play major roles later in childhood. Basing on Kliegel et al. *process model* (2002), executive processes have been further suggested to have different implications during the various phases of prospective remembering (Mahy et al., 2014).

Kliegel et al. (2002, 2011) have proposed that PM is a multi-phase process, highlighting the importance of considering the various phases of prospective remembering in studying underlying processes of PM. Specifically, their model is based on Kliegel et al.’s (2000) complex PM paradigm, which is aimed at disentangling the different phases of PM. Subsequently, the authors tested their predictions regarding which different executive processes are involved in the different phases of PM, such as planning during the intention formation phase, retrospective memory during the retention interval, monitoring, cognitive flexibility, and inhibition during the initiation and execution phases (Kliegel et al., 2002). On the basis of the process model and recent research evidence, Mahy et al. (2014) have incorporated these predictions into their model by proposing that WM and planning play critical roles during the formation and retention phases, whereas attentional monitoring, inhibitory control, and switching are relevant during the retrieval and execution phase, and thus for ongoing task performance and PM cue detection.

## Development of prospective memory and its underlying mechanisms throughout childhood

The literature on PM development has shown that PM performance increases during the preschool years (from ages 2–3 to 5–6 years; (Atance & Jackson, 2009; Kvavilashvili et al., 2001; Mahy & Moses, 2011), over the school-age period, and especially around the ages 7–8 and 10–11 years (Kerns, 2000; Spiess et al., 2016; Yang et al., 2011), until late adolescence and young adulthood (Kretschmer-Trendowicz & Altgassen, 2016; Shum et al., 2008; Ward et al., 2005; Zimmermann & Meier, 2006). Studies have shown that developmental advances differ as a function of the type of PM task, with performance on focal PM tasks improving earlier than on non-focal and time-based PM tasks (Zuber et al., 2019). Correspondingly, developmental differences in focal PM tasks are smaller than in non-focal and time-based PM tasks. This effect appears to depend on the level of cognitive resources needed to perform the various PM tasks, with focal PM tasks relying on spontaneous bottom-up processes, and non-focal and time-based PM tasks being based on strategic monitoring and top-down processes (McDaniel et al., 2015), such as attentional and WM resources (Smith & Bayen, 2005; Smith et al., 2010). For example, Zuber et al. (2019) investigated how executive functions are implicated in various PM tasks during the school-age period (6–11 years of age). WM updating was related to performance in all PM tasks, and inhibition was related to both focal and non-focal event-based PM tasks, whereas switching was relevant to performance in the non-focal and time-based PM tasks. Moreover, age-related improvements were more pronounced in non-focal and time-based PM tasks. Similarly, earlier studies of school-aged children found that WM, inhibition, and switching abilities are related to PM performance, particularly when the PM task is resource-demanding. By contrast, at earlier ages (i.e., 3–4 to 5–6 years of age), mainly WM and inhibition appear to be linked to PM performance (see Mahy et al., 2014, for a review).

The contribution of executive processes has been further studied by taking attentional monitoring costs into account (Smith, 2003; Smith & Bayen, 2004). In accordance with the executive framework of PM (Mahy et al., 2014), studies have shown that attentional monitoring costs appear around the age of 5–6 years and that the functionality of these costs (i.e., successful PM performance) increases during school-age years (Cejudo et al., 2019; Cottini et al., 2021b; Cottini & Meier, 2020; Kretschmer-Trendowicz & Altgassen, 2016; Smith et al., 2010). A recent study suggested that the use of attentional monitoring is related to advances in metacognition. Cottini et al. (2021b) found that compared to 5–6-year-olds, only 8–10-year-olds displayed attentional monitoring costs and that these costs were significantly related to performance predictions and PM performance. Performance predictions and related confidence judgments are used to measure metacognitive monitoring abilities (e.g., Roebers, 2002). Recent investigations have revealed that being able to correctly predict one’s performance or being more cautious in these judgments might be relevant for PM development (Cottini et al., 2021b).

Previous studies in the retrospective memory domain have shown that the development of metacognition, that is, knowledge about cognitive processes (i.e., declarative metacognition), and the ability to monitor, regulate, and control one’s own cognitive performance (i.e., procedural metacognition; Flavell & Wellman, 1975), becomes increasingly relevant in memory development during the school years (see Schneider, 2015). Whereas children’s knowledge of memory processes and strategies develops in the early school years, the ability to translate that knowledge into effective control (e.g., strategy use) emerges only later, around the ages of 9 or 10 years (e.g., Lockl & Schneider, 2004). Similarly, a recent study in the PM field has indicated that 6- to 13-year-old children can predict their PM performance, but only older children can effectively use external reminders (Redshaw et al., 2018). In the *meta-intentional research framework*, Smith (2016) has suggested that metacognitive monitoring and control differ in their involvement during the different phases of prospective remembering: metacognitive monitoring would be mainly relevant during the encoding phase (e.g., performance prediction, task difficulty evaluation) as well as during and after the execution phase (e.g., performance postdiction), whereas metacognitive control plays a major role during the encoding phase (e.g., planning, encoding time, and setting reminders), the retention phase (e.g., checking reminders and effectively allocating attentional resources), and the execution phase (e.g., effectively allocating attentional resources). Accordingly, the development of metacognitive monitoring and control positively affect the development of PM development (see Cottini et al., 2021b).

Another cognitive process that is likely to underlie PM development is the ability to envision the future (i.e., EFT; Atance & O’Neill, 2001). When an intention is created during the encoding phase, we might imagine the moment in future during which we carry out our intention. Recent studies found relations between EFT and PM performance in adults (e.g., Terrett et al., 2016) and children (e.g., Nigro et al., 2014). For instance, Nigro et al. (2014) have shown that EFT significantly correlates with PM performance in 7-year-olds, but not in 4- to 6-year-olds. This finding is consistent with developmental research on EFT showing that the ability to envision the future begins to develop at the age of 3 to 4 years (e.g., Atance & O’Neill, 2005; Suddendorf & Redshaw, 2013) and improves considerably during the primary school years (e.g., Coughlin et al., 2017; Ferretti et al., 2018) and adolescence (e.g., Gott & Lah, 2014). In this regard, Kvavilashvili and Rummel (2020) have argued that EFT should be integrated into current models of PM, because it is important not only during the encoding phase, when the future task is planned and envisioned, but potentially also during the retention phase, for instance, in rethinking of the future task.

## Strategies to improve children’s prospective memory performance and their effectiveness

**Fig. 1:**
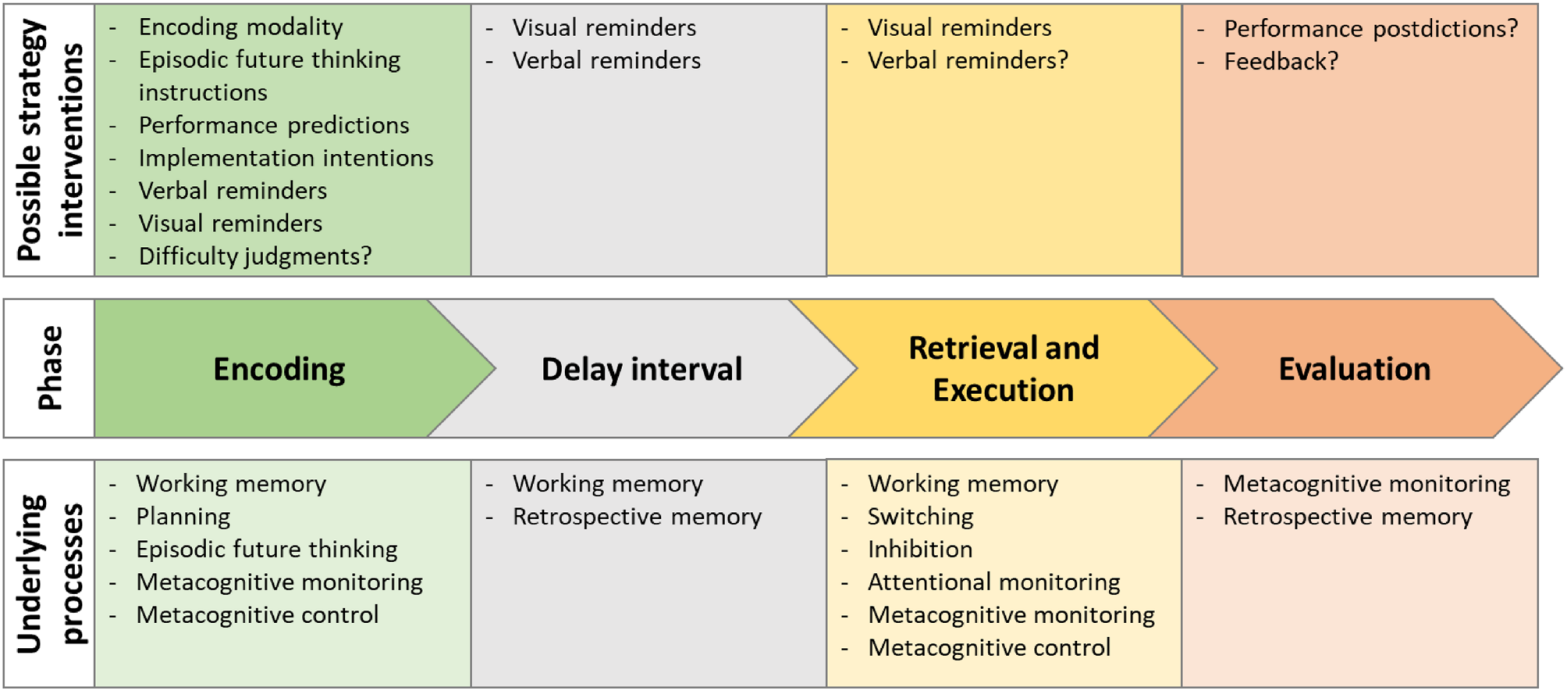
Possible strategy interventions and mechanisms underlying prospective memory during the different phases of prospective remembering. The proposed strategy interventions are based on the reviewed studies as well as on suggestions made in the literature. The latter are indicated by the question mark (?)

On the basis of the knowledge of mechanisms underlying PM development, several strategies and interventions have been proposed, developed, and evaluated in support of children’s PM over the past 50 years. Because the processes underlying PM show different involvement in the various phases of prospective remembering (Kliegel et al., 2002, 2011; Mahy et al., 2014), different strategy interventions might potentially be helpful if implemented during the different phases. Figure 1 illustrates possible strategy interventions to improve PM during the different phases of prospective remembering, on the basis of empirical findings and suggestions in the literature. Moreover, mechanisms underlying prospective remembering during the PM phases are integrated in the diagram. Empirical research findings on the effectiveness of these strategies are summarized in Tables 1 and 2 by considering the setting (i.e., laboratory vs. natural), the PM task type (i.e., event-, activity-, and time-based), the PM cue type (i.e., focal/non-focal, specific/categorical), children’s age range, the effectiveness of the strategy on improving PM performance, and the cognitive cost of remembering the PM task represented by slower RTs or higher error rates during the ongoing task (Smith, 2003; Smith & Bayen, 2004).

**Table 1:** The effects of encoding modality and encoding strategies on children’s prospective memory (PM) and ongoing task (OT) performance

Intervention	Study	Experimental setting	PM task type	PM cue type	Age	PM performance	OT performance
Encoding modality (visual, verbal, and enacted)	Passolunghi et al. (1995)—Study 1 and 2	Laboratory	Event-based	Focal and specific	7–10	Significant effect of visual (younger children) and enacted encoding (older children)	–
	Passolunghi et al. (1995)—Study 3	Laboratory	Event-based	Focal and specific	7	Significant effect of enacted encoding with cue-action association	–
	Li and Wang (2015)	Laboratory	Event-based	Focal and specific	7–9	Significant effect of target presence (younger children) and enacted encoding (older children)	No significant effect
Implementation intention	Kretschmer-Trendowicz et al. (2021)	Laboratory	Event-based	Focal and specific	9, 12,15	No significant effect	No significant effect
	Zimmermann and Meier (2010)	Laboratory	Event-based	Focal and categorical	10–14	No significant effect	No significant effect
	Yang et al. (2023)	Laboratory	Event-based Activity-based Time-based	Focal and specific	7–11	No significant effect	–
			Event-based Activity-based Time-based			No significant effect	–
			Event-based Activity-based Time-based			No significant effect (but significant PM task × WM)	–
Episodic future thinking (EFT)	Kretschmer-Trendowicz et al. (2016)	Laboratory	event-based	Focal and categorical	5–8	Significant effect	No significant effect
	Kretschmer-Trendowicz et al. (2017)	Laboratory	Event-based	Non-focal	10–12	Significant effect	No significant effect
PM predictions	Cottini et al. (2018)	Laboratory	Event-based	Focal and categorical	7–8	Significant effect	Slower OT RTs
	Cottini et al. (2019)	Laboratory	Event-based	Focal and specific	7–8	No significant effect (significant faster PM RTs)	No significant effect
EFT + PM predictions	Cottini et al., (2021a)	Laboratory	Event-based	Focal and categorical	8–11	Significant effect	No significant effect

**Table 2:** The effects of visual and verbal reminders on children’s prospective memory (PM) and ongoing task (OT) performance

Intervention	Study	Experimental setting	PM task type	PM cue type	Age	PM performance	OT performance
Visual reminder	Kliegel and Jäger (2007)	Laboratory	Event-based	Focal and specific	2–6	Significant effect	Higher OT accuracy
	Guajardo and Best (2000)	Laboratory	Event-based	Focal and specific	3–5	No significant effect	–
	Cheie et al. (2014)	Laboratory	Event-based	Focal and specific	3–7	Significant effect	–
	Ryder et al. (2022)	Laboratory	Activity-based	–	5–7	Significant effect	–
	Meacham and Colombo (1980)	Laboratory	Activity-based	–	6–8	Significant effect	–
	Meacham and Dumitru (1975)	Laboratory	Activity-based	–	6–8	no significant Effect	–
	Chen et al (2017)	Laboratory	Event-based	Focal and specific	13	Significant effect	Faster OT RTs and higher accuracy rates
Verbal reminder	Mahy et al. (2018)	Laboratory	Event-based	Non-focal and categorical	4–6	No significant effect	No significant effect

Strategies can be distinguished in internal (e.g., encoding strategies) and external (e.g., reminders) as well as based on the phase of PM in which they are deployed (i.e., encoding, retention, performance and execution phase; Ellis, 1996). Many interventions are implemented in the initial phase of PM (when the intention is generated). Interventions adopted during the encoding phase can be providing different encoding modalities (Li & Wang, 2015; Passolunghi et al., 1995) or encouraging participants to use encoding strategies such as implementation intentions and EFT or to make performance predictions (e.g., Cottini et al., 2018; Kretschmer-Trendowicz et al., 2016, 2021). By contrast, other interventions aim to relieve children from deploying cognitive resources by offering external reminders for the PM task. These are relevant after the encoding phase, during the retention, performance, and execution interval, and can include visual or verbal reminders (e.g., Kliegel & Jäger, 2007; Mahy et al., 2018).

All these strategies differently affect PM performance during childhood. In this vein, some studies have explored the mechanisms underlying these effects by considering ongoing task performance costs resulting from the strategy-use. For instance, several strategies can increase PM performance by either reinforcing the association between the PM cue and the intention or by deepening intention encoding in memory, thereby increasing the automaticity of intention retrieval. Conversely, some interventions encourage the engagement of attentional monitoring, relying on strategic retrieval processes and producing performance costs during the ongoing task (see Altgassen et al., 2016). The underlying mechanisms might shed light on developmental differences regarding the effectiveness of interventions. Each type of intervention will be described in the following sections, and its effectiveness will be discussed considering task characteristics, underlying mechanisms, and cognitive development.

## The effects of encoding modalities and encoding strategies on prospective memory performance

Studies evaluating the effects of encoding modalities and encoding strategies are summarized in Table 1.

### Verbal, visual, and motoric encoding of prospective memory instructions

To date, only two studies have evaluated the effects of different encoding modalities on children’s PM performance (Li & Wang, 2015; Passolunghi et al., 1995). Both have compared groups of children who were asked to encode PM instructions verbally, visually, or by enacting the intention. For instance, Passolunghi et al. (1995) conducted a series of experiments in which they manipulated the modality of PM instruction encoding (i.e., verbal, visual, or enacted). Primary school children were presented with a series of word lists on a computer screen and asked to press a specific key on the keyboard whenever the word “*boat*” appeared. The authors showed that 7- and 8-year-old children who were shown the visual representation of the PM target (i.e., the picture of a boat) obtained higher PM scores than their peers who were shown the written word “*boat*” during the instructions. By contrast, 10- and 11-year-old children’s PM performance benefited most when they could enact the PM intention (i.e., press the key after the PM instructions) rather than being verbally or visually presented with the PM target.

Similarly, Li and Wang (2015) showed that only 7-year-old children profited from the presence of the objects representing the PM targets (e.g., showing a mirror for the PM target “*looking in the mirror*”), while 8- and 9-year-olds benefited most from enacting the PM intention (e.g., pretending to look in a mirror or looking in a real mirror for the PM target “*looking in the mirror*”). The authors argued that 7-year-old children might still develop the cognitive abilities required to imagine the specific object while enacting the intention (see also Mecklenbräuker et al., 2011). Alternatively, they suggested that imagining the specific object during the enactment might overload young children’s still developing cognitive resources. Consequently, younger children would benefit from the object's presence to better encode an intention, as has been shown by Passolunghi et al. (1995). On the other hand, older children profit more from enacting the intention. Because no ongoing task performance costs depended on encoding modality, the authors suggested that this strategy is likely to enhance the automaticity of intention retrieval by increasing cue distinctiveness or the link between the cue and the related action (Li & Wang, 2015).

### Implementation intentions

During the encoding phase, when we form an intention and think of what we must do and when we must do it, one strategy is to rehearse and visualize the plan that must be enacted. This task can be accomplished by implementing intentions in statements that specify the plan, such as “If situation x appears, I will do y” (Gollwitzer, 1999). This encoding strategy has been shown to improve goal attainment in everyday-life tasks (Duckworth et al., 2013; Gollwitzer, 1999; Wieber et al., 2015) and PM performance (Chasteen et al., 2001; Lee et al., 2016; Zimmermann & Meier, 2010). Participants are usually required to verbally reformulate PM instructions in the typical if–then format and repeat the sentence aloud thrice (e.g., Zimmermann & Meier, 2010). Alternatively, participants were asked to visualize the PM instructions for 30–45 s (e.g., Brewer et al., 2011) or to perform the verbal and the imagery form together (e.g., McDaniel et al., 2008). A meta-analysis on the effects of implementation intention on PM performance of healthy young adults revealed that all three versions are effective; however, combining the verbal and the imagery form produced larger effects than the two formats applied alone (Chen et al., 2015). The authors also found that implementation intention enhanced PM performance in both focal and non-focal PM tasks.

Concerning the underlying mechanisms, it remains unclear whether implementation intentions elicit automatic retrieval or strategic monitoring processes. To date, three main positions have been elaborated (see Chen et al., 2015). The first postulates that implementation intention establishes a robust cue-intention association by eliciting spontaneous retrieval of the intention without bearing on cognitive resources (Brewer et al., 2011; Gollwitzer, 1999; McDaniel et al., 2008). The second assumes that implementation intention influences the PM task's importance perception, inducing participants to engage more cognitive resources to perform the task. Accordingly, a PM performance boost induced by implementation intentions would be accompanied by an ongoing task performance cost (Brewer & Marsh, 2010; Meeks & Marsh, 2010; Smith et al., 2014). The third hypothesis argues that implementation intentions would strengthen the cue-intention link and simultaneously elicit the engagement of cognitive resources (McDaniel & Scullin, 2010).

In developing populations, implementation intentions have been investigated in three studies. The first study examined the effect of the verbal version of implementation intentions on PM performance across the lifespan, comparing 10- to 14-year-olds with young and older adults (Zimmermann & Meier, 2010). Participants were required to perform a lexical decision task as ongoing task. For the PM task, they were required to press a key whenever the word of an animal was presented (i.e., focal and categorical PM cue). Implementation intentions improved PM performance across the lifespan with a significantly higher advantage for older than younger adults and a numerical but non-significant benefit for children and adolescents. Monitoring costs were similarly displayed across all three age groups and in both conditions. Consequently, implementation intentions did not further affect monitoring costs, suggesting that this strategy does not involve additional cognitive resources.

In a more recent study, Kretschmer-Trendowicz et al. (2021) investigated the impact of implementation intentions in the combined verbal and imagery format on 9-, 12-, and 15-year-old participants’ PM performance in two experiments with varying resource demands. In Experiment 1, children and adolescents received standard PM instructions or implementation intention instructions. The importance of the PM task was manipulated within participants when giving instructions to induce them to allocate their attentional resources either to the PM or the ongoing task. As ongoing activity, participants had to perform a letter comparison task in which they had to compare pairs of five-letters strings and decide whether they were equal. The PM task consisted of pressing a key when one of four specific letter strings appeared; this task was focal to the ongoing task with specific PM cues. Importance was varied across participants by saying either that it was essential to perform the PM task successfully or that high ongoing task performance was central. For implementation intentions, half of the participants were first asked to form an if–then statement for each PM cue in the first person and repeat it aloud three times. Afterward, they were requested to visualize themselves while executing the PM and the ongoing task. Neither implementation intentions nor importance of the PM task affected PM accuracy, while the importance of the PM task affected PM RTs by slowing them down. The authors argued that the missing effects of implementation intentions and importance manipulation might be due to the nature of the PM task. In fact, participants’ PM performance was quite high (i.e., near the ceiling).

In the second experiment, the authors again assigned participants either to an implementation intention or to a standard instruction condition while manipulating the task-switching demands of the ongoing task within participants. As the ongoing task, participants were required to decide whether sequentially presented black and white drawings were either living or non-living and small or big. The classification rule was specified by a cue presented jointly with the line-drawing. In the non-switching condition, participants had to classify pictures according to either one or the other criterion, whereas the classification criterion was mixed in the switching condition. The PM task consisted in pressing a key whenever two specific pictures were presented (appearing twice). Consequently, the PM task was again focal with specific PM cues. While task-switching demands negatively affected PM performance, implementation intentions had no impact on PM accuracy or PM RTs.

These outcomes contrasted with the authors’ expectations and with results obtained from previous studies including healthy young and older adult participants in which implementation intention was effective even when the PM cues were focal (Chen et al., 2015). In addition to the ease of the PM task as a possible cause for the null effect, Kretschmer-Trendowicz et al. (2021) proposed another possible explanation; they hypothesized that the lack of experience with this kind of strategy would not have permitted its beneficial effect (Einstein & McDaniel, 2007). Accordingly, children need more occasions than adults to become familiar with the strategy (i.e., metacognitive utilization deficit; Miller, 1994; Miller & Seier, 1994). This claim is supported by studies evaluating the effect of implementation intentions in children on different cognitive or non-cognitive tasks revealing contrasting results with some producing benefits (e.g., Duckworth et al., 2013) and others producing no effects (e.g., Peach & Martin, 2017). The authors also argued that, in the standard condition, children were asked to repeat the instructions several times to maintain a similar length of the PM encoding phase across conditions; this request might have similarly benefited PM performance as implementation intentions (Kretschmer-Trendowicz et al., 2021).

In response to these contrasting and limited results, a very recent study has investigated the effect of this strategy in children between 7 and 11 years of age on different PM tasks (i.e., event-, time-, and activity-based), as well as underlying processes and possible moderators of the effect (Yang et al., 2023). Participants were required to play the Fishing Game, a computerized PM task developed for children (Yang et al., 2011). While the children were asked to catch as many fish as possible (i.e., ongoing task), they were asked to click on the cat every minute to feed it (i.e., time-based task), to click on the cat whenever a striped fish appeared (i.e., focal and specific event-based task), or to press a button at the end of the game to pull the fishing boat ashore (i.e., activity-based). In line with the previously described studies including older children and adolescents, the children in the combined verbal and visualization group did not outperform those in the standard group in either PM task. However, a significant interaction between WM and PM task type, suggested that children with high WM capacity benefited from implementation intentions in the time-based PM task. The authors have argued that implementation intentions strengthen the intention and that children with higher WM are better able to keep the strengthened intention in mind and to engage monitoring resources more efficiently. Moreover, they have argued that their results are in line with those from a study by Geurten et al. (2016) showing that children’s time-based PM is predicted by metamemory only when their cognitive resources are high. Finally, the findings were also in accordance with the metacognitive utilization deficiency hypothesis, according to which both metamemory and cognitive resources are needed to efficiently use a strategy (DeMarie et al., 2004).

These three studies similarly show that implementation intentions do not appear to work effectively in children. The first two studies (Kretschmer-Trendowicz et al., 2021; Zimmermann & Meier, 2010) used focal PM tasks, whereas the third also included activity- and time-based PM tasks (Yang et al., 2023). Implementation intentions also do not appear to be effective with the latter PM tasks. However, in the time-based PM task, children with high WM appear to obtain some benefit from implementation intentions, thereby supporting the idea that this strategy might be too resource-demanding for children.

### Episodic future thinking

Like the visual format of implementation intention, EFT consists of visualizing PM instructions. It has been argued that the two encoding strategies are similar because they both require simulating the future task and involve linking the intention to the context in which it has to be retrieved (Addis et al., 2008). However, unlike implementation intentions, EFT has produced promising effects on children’s PM performance. For instance, Kretschmer-Trendowicz et al. (2016) compared a group of 5- and 7-year-old children who obtained EFT instructions with a control group of the same age. Participants were asked to name pictures depicted on cards and say the word “juice” whenever they encountered a picture of a fruit or a vegetable. This PM task was focal to the ongoing task and included a categorical PM cue, thus, it was moderately resource-demanding. Children in the EFT condition were required to close their eyes and were guided to visualize task execution. Children in the EFT group significantly outperformed children in the control group. The authors also found that 7-year-olds profited more from EFT instructions than 5-year-olds. They argued that this finding could be explained by developmental differences in EFT abilities, with older children being better able to imagine the future task and consequently benefitting more. This argument is also supported by a study by Nigro et al. (2014) showing that EFT and PM are related starting from the age of 7 years.

In a further study, investigators asked 10- to 12-year-olds to perform a complex task with real-life task demands (Kretschmer-Trendowicz et al., 2017). This task consisted of a “sight-seeing tour” in the laboratory that served as ongoing task and which required children to perform activities such as throwing marbles in a bucket or building a tower by piling small wooden sticks on the back of a wooden camel. The PM tasks were either social or neutral and required children, for example, to fill the experimenter’s glass when it was empty (social PM task) or to put on a jacket that was in another room when the experimenter opened the window (neutral PM task). Consequently, the PM tasks were non-focal to the ongoing task and were relatively resource-demanding. Again, the group who obtained EFT instructions significantly outperformed children in the control group, and this effect was independent of the nature of the PM task (neutral vs. social).

Concerning the effect of EFT instructions on ongoing task performance, results of the first study by Kretschmer-Trendowicz et al. (2016) showed that, although children’s ongoing task performance was generally worse in the block containing the PM task compared to the baseline, there were no additional monitoring costs attributable to the encoding strategy. Similarly, there were no differences between the groups regarding ongoing task performance in the second study, suggesting that EFT did not affect monitoring costs (Kretschmer-Trendowicz et al., 2017). Thus, EFT appears not to affect the amount of attentional resources engaged to perform the PM task and probably to strengthen the association between the cue and the action.

EFT appears to be a useful strategy for improving PM in children, at least starting from the age of 7 years, when the ability to envision future situations appears to be sufficiently developed (Kretschmer-Trendowicz et al., 2016). These results substantially differ from those related to the implementation intention strategy, which has been argued to be highly similar to EFT, except for the if–then statement. This difference in the effectiveness of the two strategies might be due to methodological differences among studies, such as PM task characteristics. In fact, implementation intentions have not been shown to have any effect on children’s performance on focal and specific PM tasks (Kretschmer-Trendowicz et al., 2021; Yang et al., 2023), whereas a numerical but non-significant difference has been observed when the PM task is focal and categorical (Zimmermann & Meier, 2010). Moreover, in Yang et al. ’ (2023) study, children with higher WM capacity benefitted from implementation intentions in a time-based task. Similarly, EFT efficiently enhanced children’s PM performance in a focal and categorical PM task as well as in a non-focal PM task (Kretschmer-Trendowicz et al., 2016, 2017). Because responding to focal and specific PM cues appears to require fewer executive resources than responding to categorical, non-focal, and time-based PM tasks, these strategies might be likely to be particularly efficient when the PM task is resource-demanding and when children have sufficiently developed cognitive resources to both perform the task and adopt these strategies. Finally, the if–then statement in the implementation intention strategy might involve metacognitive abilities and additionally overload children’s limited resources. Nevertheless, drawing any conclusion from the few existing studies is difficult, because no study has compared EFT with implementation intensions in children. Moreover, no studies to date have assessed the effect of EFT on children’s performance in less demanding PM tasks, including focal event-based PM targets, or the effect of implementation intentions on non-focal event-based tasks. Thus, more evidence is needed to better understand the reasons for these contrasting results.

### Performance predictions

The beneficial effect of making performance predictions was first shown in adults (Meier et al., 2011; Rummel et al., 2013). These studies found that participants who were asked to predict their PM performance outperformed participants who received standard instructions. Moreover, this positive effect was revealed when the PM task was categorical but not when it was specific (Meier et al., 2011). Rummel et al. (2013) showed that making performance judgments prior to performing a PM task also affected ongoing task performance by slowing down RTs, affecting attentional monitoring costs. Cottini et al. (2018) aimed to replicate these outcomes within a developing population. The authors asked 7-year-olds to perform a computerized picture-classification task in which they had to determine whether sequentially presented objects were part of different rooms of a house. Children had to perform the task twice with two different embedded PM tasks, one in which they were required to press a key whenever the picture of a fruit appeared (focal with categorical PM cue) and the other when three specific objects appeared (focal with specific PM cues). Participants were divided into two groups: one was asked to predict PM performance in a yes/no manner (“*Will you remember or not?*”) and to express confidence (“*How sure are you to remember/forget?*”); the control group received standard instructions. Children obtained higher PM accuracy rates when making PM performance predictions. A prediction advantage was found in the categorical but not in the specific PM task replicating the adult studies. In a subsequent study, in which a specific PM task with high WM demands was used, the authors replicated that outcome and showed that performance predictions improved RTs to the PM cues but not accuracy rates (Cottini et al., 2019).

Effects on attentional monitoring costs were found only with the categorical PM task, with children in the prediction group displaying slower ongoing task RTs than the control group (Cottini et al., 2018). To gain deeper insights into this effect, the authors conducted further analyses on the prediction group by exploring the effects of prediction accuracy and confidence judgments. While results on prediction accuracy were inconclusive, confidence judgments were negatively related to ongoing task performance slowing; children expressing lower confidence displayed higher RTs than those who were highly confident to remember the PM task. Similarly, Cottini et al. (2021b) replicated this pattern by revealing a significant negative correlation between confidence judgments and attentional monitoring costs. Marsh et al. (2005) and Smith (2016) hypothesized that when we form an intention, we predict how likely it will be that we will remember or forget. We adjust our attentional resources to execute the task based on these predictions. Whereas adults are inclined to underrate their future PM performance (Kuhlmann, 2019), children frequently overrate it (Cottini et al., 2018, 2021b; Lavis & Mahy, 2021).

In fact, children’s metacognitive monitoring abilities develop during the school years and become increasingly accurate around 10–13 years (Bertrand et al., 2017). Nevertheless, asking children to predict their PM performance appears to be effective, although they are inaccurate in their predictions. Encouraging children to reflect on their future PM performance might likely induce them to engage more attentional resources and simultaneously strengthen the encoding of the PM task. From a developmental point of view, this intervention might be more beneficial for older children who have already developed some insight into their PM ability and have more cognitive resources to engage in attentional monitoring. However, developmental studies on the effect of performance predictions, including more extensive age ranges, are lacking.

### Episodic future thinking combined with performance predictions

Starting from the evidence demonstrating the beneficial effect on PM of EFT and performance predictions, a recent study investigated the effect of combining the two encoding strategies (Cottini, 2021a). Children between 8 and 11 years were divided into standard, prediction, EFT, and combined EFT + prediction groups. After receiving encoding instructions, all children were required to perform a computerized ongoing picture-classification task. The embedded PM task consisted of pressing a key every time a picture of a fruit appeared during the ongoing task. Children in the combined encoding group obtained significantly higher PM accuracy rates than children in the single-prediction or EFT groups. This PM performance boost was not accompanied by additional attentional monitoring costs, as all groups displayed similar ongoing task performance slowing from the block without and the block with the embedded PM task.

Interestingly, when comparing children in the prediction-only with the EFT + prediction group, children in the combined encoding group who made a lower prediction had a significantly higher PM performance than those who made a higher prediction. Although this was a preliminary finding, the authors suggested that EFT might positively impact PM performance and performance predictions that, in turn, might have had an additional effect on PM performance. Nevertheless, future studies are needed to unravel these two effects and shed light on their nature.

## The effects of external reminders on prospective memory performance

Unlike interventions described in the previous sections, which are deployed during the encoding phase and require at least some of the children’s attentional, metacognitive, and EFT abilities, reminders are external strategies that are supposed to rely less on children’s cognitive resources. Various reminders have been studied in developmental research, including visual and verbal ones; these are summarized in Table 2.

### Visual reminders

Most studies investigated the effect of visual reminders, which are usually representations of the intention that must be performed placed in the participants’ sight to support their memory. One of the first studies investigating the influence of visual reminders on children’s PM was conducted by Meacham and Dumitru (1975). In this seminal work, 6- to 8-year-old children were asked to remember to place a drawing in a contest box on their way back from the experiment room to the classroom. Children were divided into three groups: a control group, a group that received a visual reminder of an envelope, and a third group that received the visual reminder and were asked to elaborate on it. In the latter condition, participants were asked how the visual reminder would help them remember and what they thought of when looking at it. Finally, they were prompted to verbalize the plan of the intention by asking to express in their own words what the visual reminder would remind them to do. Although the differences were not statistically significant between the three conditions, the results suggested that 5-year-olds remembered more frequently in the elaboration condition (57%) than in the reminder (31%) or the control condition (21%). On the other hand, PM performance was similar between conditions for 7-year-old children, who generally outperformed 5-year-olds.

Because the study was limited by a small sample size, a filler task that might have been too demanding for 5-year-olds, and a PM task that was not sufficiently motivating for children, a subsequent study was conducted to follow up on the effectiveness of reminders (Meacham & Colombo, 1980). The PM task consisted of reminding the experimenter to open a surprise box set out of the children’s view after completing an activity. Half of the 5- to 7-year-old children were engaged in the demanding filler task adopted in the previous study by Meacham and Dumitru (1975), and half played cards with the experimenter. Children were divided into a control group, and a group presented with a visual reminder and asked to elaborate on it. As a visual reminder, the experimenter showed a clown puppet placed in front of the children and required them to say what they thought about when looking at the clown. While the filler task had no effects, the children in the elaboration condition remembered significantly more frequently than children in the control group. The authors concluded that visual reminders combined with instructions to use them would be an effective intervention to help children remember.

Subsequent investigations reported similar benefits of visual reminders in children of various age groups (Cheie et al., 2014; Chen et al., 2017; Kliegel & Jäger, 2007; although see Guajardo & Best, 2000, for non-significant effects). Cheie et al. (2014) showed that providing 3- to 7-year-old children with a visual reminder of the PM target placed alongside the computer was advantageous for their PM performance. Similarly, Kliegel and Jäger (2007) showed that placing a box and an actual apple in front of 2- to 6-year-old participants increased the frequency of remembering to place apple cards (embedded in an ongoing card-naming task) in a box. The external reminder appeared to be more helpful for 3-year-olds than for older children. However, it was unclear whether the beneficial effect was due to the target (i.e., apple) or the action reminder (i.e., box) because there was no condition including either of the two reminders. In this vein, Ryder et al. (2022) tested the effect of various incidental visual reminders on an activity-based PM task. The PM task consisted in placing a card depicting a dog into a box, which was placed behind the child (i.e., out of sight) every time an activity book was completed. Each activity book included a visual search task, which required the children to name a series of pictures and simultaneously search for a specific picture (i.e., ongoing task). The incidental reminders were placed at the end of each activity book and could be the target picture (i.e., a dog), an associative picture (i.e., a cat), an action reminder (i.e., a box), or a neutral picture (i.e., a flower), which served as control. The 5- to 7-year-old children who received a PM target reminder significantly outperformed the control group, the group who received an associative reminder, and the group who received an action reminder. On the other hand, children in the latter two groups did not differ from one another in their PM performance and performed only marginally better than the control group.

Effects of reminders on monitoring costs were considered only in two studies (Chen et al., 2017; Kliegel & Jäger, 2007). In Kliegel and Jäger’s study (2007), children in the reminder group had significantly higher ongoing task accuracy rates than children in the control group, suggesting that the external reminder benefited PM and ongoing task performance. The authors argued that the memory aid was so efficient that children had even more resources to perform the ongoing task. Similarly, Chen et al. (2017) found that a PM target reminder benefited 13-year-old’s PM performance and reduced ongoing task interference effects. Adolescents in the reminder condition had higher accuracy rates and faster RTs to the ongoing task. Moreover, this beneficial effect was higher when the WM load within the ongoing task was high compared to when it was low, suggesting that the reminder improved PM performance and reduced monitoring costs even under a high WM load. These results suggest that external reminders could effectively enhance PM performance by releasing children from engaging cognitive resources. These reminders would be highly beneficial especially for young children with still developing cognitive abilities.

### Verbal reminders

Unlike studies of visual reminders, studies of verbal reminders are scarce. There is only one study to date in which the impact of explicit verbal reminders on children’s PM performance has been investigated. Mahy et al. (2018) asked 4- to 6-year-old children to remember to place animal cards in a box while they were engaged in an ongoing activity consisting in naming the location of a red sticker on the cards. Children were allocated to a control group, a retrospective reminder group (in which children were reminded of the PM task instructions before beginning the task), and an executive reminder group (in which children were reminded to pay attention to the cards). Although PM performance increased with age, neither of the reminder types significantly improved PM performance.

On the contrary, in 4-year-olds, the executive reminder produced a negative effect, with children in this group performing worse than peers in the other two groups. The authors argued that 4-year-olds might have been distracted by the executive reminder, that they might have interpreted it as an instruction to pay attention to the ongoing task, or that they did not benefit from the reminder because of a lack of cognitive and metacognitive abilities. Furthermore, the authors claimed that the missing effect of explicit verbal reminders might have been due to several factors: verbal reminders might be effective only if they are given during the ongoing task shortly before the presentation of the PM cue; children might have ignored the verbal reminder because they were used to this type of directions by adults in everyday life, or verbal reminders were not salient enough compared to visual ones. Future studies are needed to test these hypotheses and explore verbal reminders for various PM tasks. Moreover, it would be essential to explore the effect of verbal reminders in older children who have developed the necessary cognitive and metacognitive abilities and therefore might take advantage of these types of reminders.

## Which are the most effective strategies for improving children’s prospective memory performance?

Several interventions and strategies to improve PM performance in children have been addressed. In the last 50 years, studies have investigated the effects of (1) encoding modalities such as verbal, visual, and motoric encoding of PM instructions, (2) encoding strategies such as implementation intentions, EFT, and performance predictions; and (3) external reminders such as visual and verbal reminders.

To summarize, the only two studies that evaluated ways to encode PM instructions revealed consistent age-related effects (Li & Wang, 2015; Passolunghi et al., 1995). Verbal encoding of instructions appears to be ineffective for younger and older children, whereas visual encoding appears to be helpful for younger children and enacting PM instructions for older ones. These different effects might be due to the development of the processes involved in the different encoding modalities. Enacting the future task might involve WM as well as more complex EFT abilities, which have yet to develop in younger children. Thus, including age-appropriate encoding modalities when PM instructions are given (i.e., visual for younger children and motoric for older children) is likely to support children’s visualization of the future task and strengthen the intention. However, both studies evaluated PM performance in laboratory-based experiments by adopting event-based PM tasks with focal and specific PM cues. Further studies are needed to determine whether providing children with visual representations of intentions or letting them enact prospective tasks would improve performance also in PM tasks that are resource-demanding or non-focal.

In addition to encoding modalities, the present review outlined the effects of different encoding strategies. Among these, encouraging children to visualize the PM task in advance (i.e., EFT) or asking them to predict their PM performance boosts PM performance in younger and older children (Cottini et al., 2018; Kretschmer-Trendowicz et al., 2016, 2017). Moreover, combining EFT instructions with the request to predict PM performance further increases children’s performance (Cottini et al., 2021a). The effect of EFT appears to bear on automatic retrieval processes; performance predictions appear to foster the engagement of attentional monitoring resources. However, because making performance predictions might also involve EFT (cf. Kvavilashvili & Ford, 2014), beyond triggering attentional monitoring during the ongoing task, it might also be postulated that performance predictions additionally strengthen the cue-intention association. This might be supported by the fact that also children whose metacognitive monitoring abilities were not completely developed benefitted from making performance predictions. Moreover, the engagement of attentional monitoring prompted by performance predictions appears to be related to the development of metacognitive abilities in children (Cottini et al., 2021b). Thus, more developed metacognitive abilities would permit encoding strategies to improve PM performance. Accordingly, children younger than 7–8 years might take less advantage of performance predictions, whereas older children might gradually benefit. Similarly, EFT instructions appear to be more advantageous as the ability to visualize the future develops (Kretschmer-Trendowicz et al., 2016). Forthcoming studies should investigate the relation between age-related advances in metacognition and EFT and their effects on PM development. The effects of EFT and performance predictions should also be evaluated in non-focal PM tasks. Given the potentially positive impact of metacognitive skills and EFT abilities on PM development, future studies should also evaluate these interventions in natural settings.

Unlike EFT and performance predictions, implementation intentions did not affect PM performance in children between 7 and 15 years (Kretschmer-Trendowicz et al., 2021; Yang et al., 2023; Zimmermann & Meier, 2010). It is not clear why this encoding strategy, which is quite effective with adults (Chen et al., 2015) and highly similar to EFT, did not positively affect children’s PM performance. One plausible reason may be that implementation intention requires more cognitive and metacognitive resources than simply imagining execution of the task (cf., Yang et al., 2023). Thus, children’s resources might be overloaded by the additional if–then statement, although this possibility remains to be confirmed by future studies. Forthcoming studies should evaluate the impact of implementation intentions, for example, by (1) including a wider age range and comparing children with adults; (2) using PM tasks varying in their resource demands and cue focality; (3) considering ongoing task performance costs to shed light on underlying mechanisms of the effect, and (4) comparing implementation intention with EFT instructions that are thought to be very similar encoding strategies but have revealed differential effects on children’s PM performance. Moreover, future studies comparing different strategies across conditions would be interesting and informative. For instance, implementation intentions, performance predictions, EFT, and enactment of PM instructions all appear to share similar processes. In fact, they all involve anticipation of the intention by either imagining or enacting. From a memory research perspective, specifically according to the dual-coding theory, these strategies have been hypothesized to strengthen the memory trace and to increase the probability of remembering (Paivio, 1971). However, some of these strategies, such as performance predictions and implementation intentions, appear to rely on additional cognitive and metacognitive resources. In contrast, EFT and visual PM instructions appear to strengthen intention encoding by involving fewer cognitive resources. Forthcoming studies should shed light on the underlying mechanisms needed to adopt the different strategies.

Finally, visual reminders have substantial potential to help children remember intentions (Cheie et al., 2014; Chen et al., 2017; Kliegel & Jäger, 2007; Meacham & Colombo, 2000; Ryder et al., 2022; although see Guajardo & Best, 2000, and Meacham & Dumitru, 1975, for non-significant effects). This intervention is helpful across childhood, from 2 to 13 years. Moreover, the only two studies that examined underlying mechanisms showed that using visual reminders improved PM and ongoing task performance, suggesting that automatic retrieval of the PM task is triggered. This finding supports the great potential of this strategy in relieving children from engaging cognitive resources to perform PM tasks. However, all studies testing visual reminders used focal event-based PM tasks with specific PM cues that are known to bear less on attentional monitoring processes than, for example, time-based or non-focal PM tasks. Consequently, more studies are needed to confirm the relieving effect of visual reminders. Unlike studies of visual reminders, the only study on verbal reminders found no positive effects (Mahy et al., 2018). Given that the verbal reminder was given only once during the retention interval, it would be interesting to explore whether giving reminders during the ongoing task would have effects similar to visual reminders. However, as shown in studies evaluating the effects of encoding modalities, children are also likely to have difficulties in verbally encoding intentions (Li & Wang, 2015; Passolunghi et al., 1995). Verbal reminders are frequently used in everyday life (Mazachowsky et al., 2021); thus, more research is needed to study their effectiveness in different situations.

Although many of these interventions have improved children’s PM performance, more studies are needed to affirm which are the most powerful ones to enhance PM performance during different age periods. Importantly, not all interventions have been evaluated across all age groups; thus, it is unclear whether children of different ages would similarly benefit from the several interventions. Moreover, some potentially beneficial strategies have not been evaluated, such as asking children to judge the difficulty of a PM task beforehand or asking them to judge performance after a PM task or giving them PM performance feedback. For instance, a recent study has shown that difficulty judgments are related to attentional monitoring costs, with children expecting a PM task to be difficult engaging more attentional resources during the ongoing task (Cottini et al., 2021a). However, no study to date has evaluated the direct effect of making difficulty judgments on PM performance by comparing two groups of children with and without difficulty judgments. Similarly, regarding the post-performance phase, which has been widely neglected in the PM literature, performance postdictions or performance feedback presumably might affect future PM performance. For example, correctly judging one’s past PM performance has been argued to contribute to more efficient engagement of strategic behavior with future PM tasks (see Meeks et al., 2007). Nevertheless, no study to date has evaluated the effects of performance postdictions or performance feedback on future PM performance.

## Linking the effects of prospective memory interventions to theoretical frameworks

As hypothesized in different models of PM (Kliegel et al., 2002; Kvavilashvili & Rummel, 2020; Mahy et al., 2014; Smith, 2016), during the encoding phase, various abilities are involved, such as WM, planning, EFT, and metacognitive monitoring and control. Subsequently, during the retention interval, WM and retrospective memory processes are used. In contrast, depending on the characteristics of the PM and the ongoing task (Einstein & McDaniel, 2005; McDaniel et al., 2015), during the retention and execution interval, executive processes (i.e., WM, switching, inhibition, and attentional monitoring), as well as metacognitive monitoring and control, are involved. The different interventions that can be applied during those phases, and that have been tested in children, appear to differently support PM performance at different ages relying on either one or the other ability.

From a developmental point of view, the executive framework of PM development (Mahy et al., 2014) might be able to partly explain why some strategies work better at specific ages, whereas others are less efficient. Accordingly, retrospective memory is mainly relevant in early years, while the development of executive functions drives the development of PM starting from the age of 4 years, with different executive functions being more or less relevant at different ages and during the different phases of PM (see also Kliegel et al., 2002). However, beyond executive functions, the model should be integrated with additional important driving forces of PM development. These driving forces include EFT and metacognition, which have been shown to be relevant in PM development, particularly starting from primary school (Cottini et al., 2021a, 2021b; Kretschmer-Trendowicz et al., 2016). As discussed in the previous section, although EFT might be supportive for PM development earlier in childhood, this strategy might be increasingly beneficial starting from the age of 7 years (Kretschmer-Trendowicz et al., 2016). Notably, during the primary school years, important advances in episodic memory and executive functions occur (Shing & Lindenberger, 2011; Zelazo et al., 2004), which appear to be relevant for the development of both EFT and PM (Atance & O’Neill, 2005; Mahy et al., 2014). Similarly, metacognition has been argued to gradually support PM development during the primary school years, not only thanks to advances in metacognitive knowledge, but also thanks to the development of episodic memory and executive functions (Schneider, 2015). Finally, considering the phases of prospective remembering addressed in the executive framework, EFT and metacognition might play a major role not only during encoding but also in the following phases of PM (Kvavilashvili & Rummel, 2020; Smith, 2016).

According to developmental studies and theories related to metacognition, the ability to efficiently use strategies to enhance memory performance advances through different stages (Flavell et al., 1966; Moely et al., 1969; Roebers, 2014, for a review). First, young children frequently appear to experience a mediation deficiency; that is, they know the strategy to use, but they have not yet developed the required cognitive resources to execute the learned or instructed strategy. Then, during the school years, children often experience a production deficiency; that is, they know the strategy and have developed the required cognitive resources to adopt it, but they do not use it spontaneously, probably because they have insufficient experience of its usefulness. After that stage, children might experience utilization deficiency; that is, they know and are able to execute the learned strategy, but their performance is not as expected, either because its use is not yet automized or because children lack the sensitivity to know when and how the strategy is most effective. Consequently, being able to efficiently adopt a strategy to improve performance might depend not only on the knowledge of that strategy but also on the development of cognitive resources necessary to engage the strategy and on experience in using the strategy. This aspect might explain the inconsistent results and developmental differences reported in the effectiveness of some strategies (e.g., EFT and implementation intention), thus further supporting the claim that considering metacognition, beyond EFT and executive functions, within the framework of PM development is important. This consideration would represent the first step toward a more comprehensive framework of PM development which includes executive processes, retrospective memory, EFT, and metacognition.

## Implications for everyday life and conclusions

Most prior studies have been conducted in laboratory-based settings, and only a few took place in a natural setting, such as at home or in school. As recently highlighted by Rummel and Kvavilashvili (2021), PM requirements in laboratory settings are quite different from those in the real world. In fact, ecological studies conducted in natural settings have not always confirmed findings from the laboratory (e.g., Schnitzspahn et al., 2011; Unsworth et al., 2012). Beyond the higher complexity and unpredictability of the real world, the delay period between intention formation and execution is usually much longer in real life than in laboratory-based tasks (Rummel & Kvavilashvili, 2021). For instance, the real-life PM performance of young children, between 2 and 4 years of age has been shown to get worse after longer retention intervals (i.e., 5-min vs. 4- to 8-h delay; Somerville et al., 1983). Similarly, in a laboratory-based study, 4-year-olds have been found to struggle to perform the PM task after longer delay intervals (i.e., 1- vs. 5-min delay; Mahy & Moses, 2011). In contrast, 5- and 6-year-old children’s PM performance appears to benefit from long delay intervals, thus suggesting that they might be able to remind themselves of the intention or to better encode the intention (Mahy, 2021). Nevertheless, strategy interventions that have successfully improved children’s PM in laboratory settings might have different effects in the real world. Consequently, research on the applicability of these strategy interventions in natural settings is fundamental to improve their ecological validity and to make these strategies useful for everyday life, particularly in educational practice. Parents and teachers support children’s remembering differently according to age in daily life. Although adults might provide aid to preschool children in remembering intentions, school-aged children are frequently requested to remember independently (Hajdas et al., 2021). Providing visual rather than verbal reminders would be helpful for teachers and parents to support preschoolers’ and older children’s remembering. Moreover, younger children might benefit from being shown a visual representation of the intention, for example, a picture of the object or the object itself, if they are asked to remember to bring that object to school. For instance, teachers can provide children with a sticker or a card depicting the letter that the children need to deliver to their parents after they go home.

Conversely, when entering primary school, it might be essential to support children’s remembering of intentions by teaching them to use different strategies and guiding and train them to become progressively autonomous. For example, learning to visualize the to-be-remembered activities or to monitor their own PM performance by predicting and reflecting it might be helpful for school-aged children, particularly starting from the age of 7 or 8 years. For instance, teachers might train children to visualize themselves doing their homework in the afternoon or delivering a message to their parents after going home. Similarly, teachers might help children reflect on their own PM performance and let them predict their probability of remembering autonomously to bring a book to school the next day. Moreover, they might help children judge the difficulty of remembering and reflect on different strategies that they could use for different PM tasks. To accomplish this goal, teachers and parents should know appropriate strategies to teach from a developmental perspective in order to avoid children’s frustration and maximize their learning and remembering. Moreover, according to the developmental stages of procedural metacognition (Flavell et al., 1966; Moely et al., 1969; Roebers, 2014, for a review), after children have developed the necessary cognitive resources to use a particular strategy, training this strategy and letting children experience its effectiveness are important. However, a topic that must be examined in future research is identifying the most effective strategies that children can use at different ages to remember intentions in everyday life.

To conclude, the present review was aimed at outlining effective strategies identified by scientific community for improving children’s PM performance across childhood, and describing gaps and limitations in the literature to support future research. Developing the ability to remember intentions is an essential task during childhood that affects children’s autonomy and well-being in various areas of their life (e.g., school, social relationships, and health). It is hoped that this review might stimulate future investigations to better understand the effectiveness of PM strategies and interventions from a developmental perspective and to make these outcomes useful for educational practices by increasing their ecological validity.

## Data Availability

For this manuscript, any datasets were analyzed or generated, because this work proceeds within a theoretical approach.
